# Aquatic plants and ecotoxicological assessment in freshwater ecosystems: a review

**DOI:** 10.1007/s11356-020-11496-3

**Published:** 2020-11-26

**Authors:** Simona Ceschin, Amii Bellini, Massimiliano Scalici

**Affiliations:** Department of Sciences, University of Roma Tre, Viale G. Marconi, 446 00146 Rome, Italy

**Keywords:** Freshwater plant organisms, Ecotoxicology, Plant-based ecotoxicological test, Toxicant, Water contamination, Aquatic ecosystem

## Abstract

This paper reviews the current state-of-the-art, limitations, critical issues, and new directions in freshwater plant ecotoxicology. We selected peer-reviewed studies using relevant databases and for each (1) publication year, (2) test plant species, (3) reference plant group (microalgae, macroalgae, bryophytes, pteridophytes, flowering plants), (4) toxicant tested (heavy metal, pharmaceutical product, hydrocarbon, pesticide, surfactant, plastic), (5) experiment site (laboratory, field), and (6) toxicant exposure duration. Although aquatic plant organisms play a key role in the functioning of freshwater ecosystems, mainly linked to their primary productivity, their use as biological models in ecotoxicological tests was limited if compared to animals. Also, toxicant effects on freshwater plants were scarcely investigated and limited to studies on microalgae (80%), or only to a certain number of recurrent species (*Pseudokirchneriella subcapitata*, *Chlorella vulgaris*, *Lemna minor*, *Myriophyllum spicatum*). The most widely tested toxicants on plants were heavy metals (74%), followed by pharmaceutical products and hydrocarbons (7%), while the most commonly utilized endpoints in tests were plant growth inhibition, variations in dry or fresh weight, morpho-structural alterations, chlorosis, and/or necrosis. The main critical issues emerged from plant-based ecotoxicological tests were the narrow range of species and endpoints considered, the lack of environmental relevance, the excessively short exposure times, and the culture media potentially reacting with toxicants. Proposals to overcome these issues are discussed.

**Supplementary Information:**

The online version of this article (10.1007/s11356-020-11496-3) contains supplementary material, which is available to authorized users.

## Introduction

Ecotoxicological studies have increased significantly in the last decades following the exponential growth in the production and use of chemicals in agriculture, medicine, and various industrial sectors, leading to an increasing release of toxic contaminants into waters globally (Paixao et al. 2008; Decou et al. 2018; Ma et al. 2019). In particular, inland waters are among the most threatened habitats worldwide by this indiscriminate pollution (Reid et al. 2019) and their protection ought to be part of the priorities of environmental conservation and management policies, considering both the peculiarity of their biocenoses and the fundamental role that these habitats assume for humans as a direct source of cultural and socio-economic resources, activities, and ecosystemic services (Aylward et al. 2005; Pham et al. 2019). Consequently, monitoring activities of water quality and health environmental status assume considerable importance and, from this perspective, ecotoxicological studies play an evident role in assessing contaminant effects on aquatic habitats and human health (Paixao et al. 2008; Mkandawire et al. 2014).

Nowadays, government policies aim at estimating and monitoring the impact of detrimental chemicals on both environmental and human health by using diverse strategies involving toxicological and ecotoxicological tests, developed to provide suitable tools for analyzing negative effects of toxicants. Through legislations, policy makers may provide new tools to researchers and institutions which are interested in preserving the quality of the environment, for evaluating and mitigating the effects of pollutants on the ecosystems, such as the Water Framework Directive (WFD; 2000/60/EC). Following this directive, in order to detect environmental hazards and potential risks to human health, the employment of sentinel species as early environmental warning systems is strongly encouraged. In fact, traditional water chemical analyses combined with biological monitoring activities allow an integrated and more complete assessment of the water quality status.

In this context, plant-based ecotoxicological tests assume an evident role, considering that many contaminants enter the ecosystem through the plant organisms (i.e., autotrophic organisms) that are the first and obligate step of the main trophic chains. Thus, plants can accumulate toxicants, and herbivores will be contaminated with the potential of food chain contamination by bioaccumulation and biomagnification processes. Among the plant models that may be exploited in ecotoxicological investigations in freshwater ecosystems, there is a wide variety of aquatic plant organisms ranging from algae, bryophytes, pteridophytes, and flowering plants. These usually are utilized as bioindicators of water quality status (e.g., Ceschin et al. 2010, 2012; Søndergaard et al. 2010; Wu et al. 2017), and of hydro-morphological alterations of river and lake ecosystems (e.g., Daniel et al. 2006; Benítez-Mora and Camargo 2014; Tombolini et al. 2014; Abati et al. 2016), as well as phytoremediation agents for the removal of toxicants from civil, agricultural, and industrial wastewater (e.g., Tripathi and Shukla 1991; Mkandawire et al. 2014; Li et al. 2018; Ceschin et al. 2019, 2020). In the first case, the sensitivity of some plant species to certain contaminants or alteration conditions is exploited, while in the second case, the focus is on the capability of other species to tolerate the presence of contaminants and bioaccumulate them in their tissues. These properties, recognized in various freshwater plant organisms, lend themselves considerably to be exploited also in the ecotoxicological sector when evaluating the contamination status of freshwaters.

On the basis of the above mentioned, it appears surprising that the use of aquatic plants as biological models in ecotoxicological tests is still rather limited (Blinova 2004; EPA 2020); in fact, the most frequently used organisms for these tests are bacteria (e.g., *Pseudomonas putida*) (Sihtmäe et al. 2013) and, especially, animals such as aquatic micro- and macroinvertebrates (e.g., *Daphnia magna*, *Gammarus duebeni*) (Chae et al. 2018; Mateos-Cárdenas et al. 2019) and fish (e.g., the Mozambique tilapia, *Oreochromis mossambicus* and the zebrafish, *Danio rerio*) (Xue et al. 2018; Godoy et al. 2019). When it comes to the toxicological effects of various contaminants on the different aquatic plant organisms, the knowledge is equally scarce (Coutris et al. 2011; Alkimin et al. 2019), with most of the available studies focusing mainly on unicellular algae (Wang 2019).

The neglected use of freshwater plants with respect to animals could be due to the idea that the former were considered less sensitive to chemicals (Lewis 1995). Furthermore, traditionally toxicological studies have focused primarily on the evaluation of the toxic potential of contaminants present in water with particular reference to human health and, from this perspective, plant organisms have often been considered unsuitable biological models for such evaluations, since they are biologically too distant from the human organism (Lewis 1995; Blinova 2004), and therefore less useful, apparently, in assessing the possible influence of a contaminant on its well-being.

Anyhow, ecotoxicological studies in freshwaters based on plant organisms become fundamental tools to evaluate and monitor the quality and health status of both human beings and aquatic ecosystems, since plant organisms play a structurally and functionally fundamental ecosystemic role as primary producers (Gubbins et al. 2011); suffice it to say that if plant organisms bioaccumulate toxicants present in water, first the herbivores and then the carnivores will be contaminated along the food chain following a biomagnification process that can finally lead to the man at the top of this process of contamination. Hence, plant-based ecotoxicological studies have the potential to identify possible toxicological risks in the environment, since contamination phenomena recorded in the plants directly/indirectly affect all the other organisms and the health of the entire ecosystem, including humans (Geis et al. 2000; Costa et al. 2018). In addition, being the first interface between abiotic and biotic components of an ecosystem, plant organisms can respond to water contamination phenomena earlier than other organisms, assuming the important role of early warning systems (EWSs), which is fundamental for intercepting contaminations in advance and allowing a timely intervention before the processes of biomagnification along food chain or the diffusion of the contaminant become too advanced.

An encouragement in using plants in ecotoxicological studies emerges from an analysis of the Toxic Substances Control Act (TSCA) for the premanufacturing notifications of chemical substances that highlighted how freshwater unicellular algae and animals show a different sensitivity to various contaminants (Lewis 1995). Therefore, in order to exhaustively evaluate the toxic effect of a contaminant in the environment, it is important to carry out ecotoxicological tests, not only on animal organisms, but also on plants (Coutris et al. 2011; Alkimin et al. 2019).

Within this context, the present paper reviews the current state-of-the-art in the use of aquatic plant organisms in ecotoxicological investigations in freshwater ecosystems. The information extracted from the literature was summarized and evaluated by considering (i) the proportion of ecotoxicological studies dedicated to plant organisms and different groups of plants; (ii) the different plant-based ecotoxicological tests available; and (iii) the main toxicants employed in these tests. In addition, limitations and critical issues in freshwater plant ecotoxicology are highlighted, and proposals to overcome these issues are discussed.

## Methodology

### Information sources

International scientific articles were selected from various sources, such as Scopus, Web of Science, Google Scholar (1900–February 2020) and, especially, ECOTOX database (EPA 2020) (1915–February 2020). Research keywords primarily included “ecotoxicology,” “freshwater plants/aquatic plants/macrophytes/hydrophytes,” “algae,” “unicellular algae,” combined with “toxicants in freshwater ecosystems,” “ecotoxicological effects/impacts/toxicity,” and various “toxicants,” such as “heavy metals, pharmaceutical products, hydrocarbons, pesticides, surfactants, plastics”.

### Search criteria

The search criteria used to screen literature were (i) articles in peer-reviewed international journals and contributions in books, excluding congress proceedings and unpublished dissertations, (ii) studies published in English language, (iii) studies reporting empirical research, i.e., referring to real data and analysis, (iv) plant-based ecotoxicological studies, and (v) experimentations in both laboratory and field conditions.

### Data collection

All extracted and selected papers were utilized for elaborating a digital database where data were grouped according to six main items: (1) publication year of the article, (2) scientific name of the test plant species, (3) reference plant group, (4) toxicant and toxicant category (heavy metal, pharmaceutical product, hydrocarbon, pesticide, surfactant, plastic), (5) test site (laboratory, field), and (6) toxicant exposure duration. As for the reference plant groups, plants were listed as microalgae, including unicellular algae (mainly diatoms, chrysophyceans, cryptomonads, unicellular green algae), macroalgae (pluricellular and thallophytic green/yellow/red algae, charophyceans), aquatic bryophytes (mosses, liverworts, hornworts), aquatic pteridophytes, and flowering plants.

## Results and discussion

### Freshwater plant organisms used in ecotoxicology

**Fig. 1:**
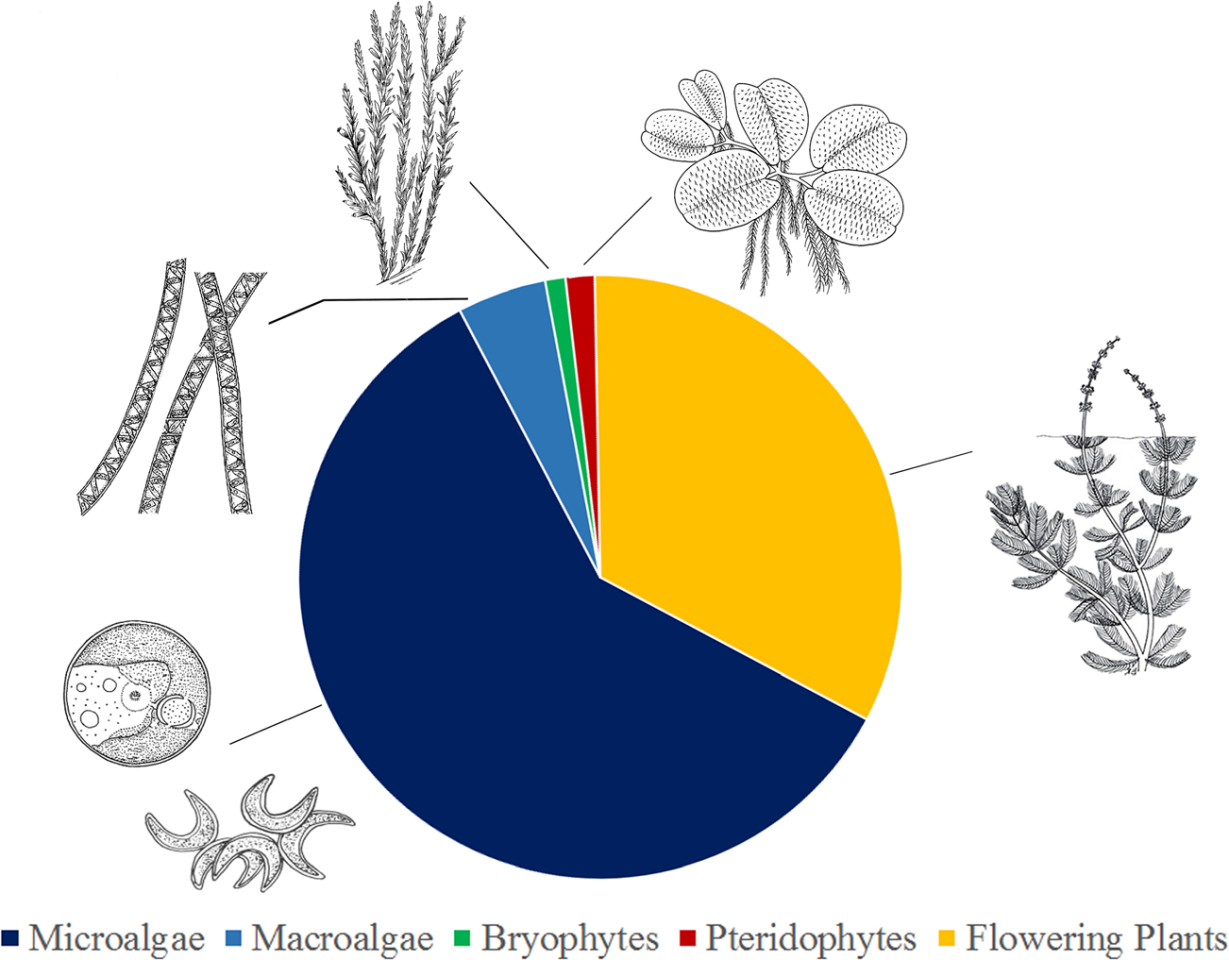
Frequency (%) of use of taxa belonging to the main plant groups (microalgae, macroalgae, pteridophytes, bryophytes, flowering plants) in ecotoxicological studies (data extracted by ECOTOX database, EPA 2020)

The main output emerging after the systematic analysis of the ECOTOX database regarding over 6000 aquatic species used in water ecotoxicology (including animals, plants, and fungi), is that only 25% are plant species, and most of these are microalgae (60%), followed by flowering plants (33%), macroalgae (around 6%), pteridophytes (1.6%), and aquatic bryophytes (1%) (Fig. 1). About 65% of the aquatic plant species used refer to freshwater plant organisms, while the remaining percentage consists of seawater and brackish taxa.

Several evidence showed that, compared to other aquatic plant organisms, microalgae are often more sensitive to certain contaminants found in civil and industrial wastewaters (Lewis 1995; Blinova 2004; Paixao et al. 2008). This would explain the reason why, among the different plant groups, microalgae were more widely used as reference species in phytotoxicological tests (Lewis 1995). In fact, microalgae-based tests were designed to be used with various types of potential toxicants and effluents, and were the first to be developed and standardized since the 1960s by regulatory development agencies, such as the Organization for Economic Cooperation and Development (OECD) and the International Standards Organization (ISO). Afterwards, starting from these tests, several research projects have tried to optimize the procedures and perform microscale tests. In fact, microalgae are suitable for performing miniaturized tests which have the economic advantage of analyzing the toxicity of a wide range of chemicals and water samples at reduced costs, as well as requiring small sample volumes for the analyses, allowing a large number of samples to be tested at the same time. Some microalgal species were used more than others in ecotoxicology studies for testing a wide range of contaminants, such as *Pseudokirchneriella subcapitata* (= *Selenastrum subcapitata*) and *Chlorella vulgaris* (Table 1).

**Table 1:** For each plant group (microalgae, macroalgae, bryophytes, pteridophytes, flowering plants), the species which are most used in ecotoxicological tests are shown. For each species, in addition to the scientific name, the number of ecotoxicological studies in which the species was tested, and the time range (year min and max) in which these studies were carried out are reported (data extracted by ECOTOX database, EPA 2020)

Plant group	Scientific name	N. paper	Year min	Year max
Microalgae	*Pseudokirchneriella subcapitata*	489	1973	2019
	*Chlorella vulgaris*	251	1962	2018
	*Chlamydomonas reinhardtii*	167	1970	2018
	*Chlorella pyrenoidosa*	167	1952	2019
	*Scenedesmus quadricauda*	164	1959	2018
Macroalgae	*Cladophora glomerata*	15	1970	2005
	*Oedogonium cardiacum*	25	1973	1986
	*Spirogyra* sp.	20	1931	2010
	*Chara* sp.	22	1960	2007
	*Zygnema* sp.	5	1963	2009
Bryophytes	*Fontinalis antipyretica*	20	1978	2013
	*Platyhypnidium riparoides*	9	1987	2009
	*Vesicularia dubyana*	4	1989	2005
	*Scapania undulata*	4	1983	1999
	*Fontinalis dalecarlica*	2	1984	1998
Pteridophytes	*Azolla pinnata*	15	1979	2005
	*Salvina molesta*	15	1975	2018
	*Azolla caroliniana*	10	1982	2016
	*Salvinia natans*	10	1972	2019
	*Azolla filiculoides*	7	1982	2013
Flowering plants	*Lemna minor*	254	1954	2019
	*Myriophyllum spicatum*	115	1963	2015
	*Hydrilla verticillata*	105	1960	2018
	*Lemna gibba*	93	1971	2017
	*Myriophyllum sibiricum*	9	1967	2005

With regard to other plant groups, since the 1950s, most tests was conducted on freshwater species of the genera *Lemma* (floating rootless flowering plants) and *Myriophyllum* (rooted flowering plants) (Table 1). The latter was used almost exclusively to determine the toxicity of sediments contaminated by various toxicants, mainly heavy metals and pharmaceutical products. It is only since the 1970s that some aquatic bryophytes (e.g., *Fontinalis antipyretica*) and pteridophytes (e.g., *Azolla pinnata*, *Salvina molesta*) were also used in phytotoxicological tests, although their frequency was much lower than the one recorded for other plant groups (Table 1).

### Plant-based ecotoxicological tests

There are various ecotoxicological tests that use plant organisms to determine the toxic effects of commercial and agricultural chemical products or industrial and civil derivatives. The use of plant-based tests started to be relatively significant only in the last two decades (Razinger et al. 2007; Alkimin et al. 2019; Dumont et al. 2019; Eagles et al. 2019), when plants have been most acknowledged as useful biomonitors in environmental toxicology. This led to an increasing number of environmental directives and regulations requiring the development of specific methodological guidelines for these tests and for their standardized use (Lewis 1995; Brain and Cedergreen 2008; Mkandawire et al. 2014). As a result, some international and national regulatory agencies, in particular the ISO and OECD, developed specific guidelines for carrying out ecotoxicological tests and standardized the methodological procedures, allowing valid comparisons among the various experimentations on the toxic effects of contaminants on freshwater plants utilized as biological models. The methodologies suggested by ISO and OECD tests differ slightly in design but all use similar plant species which are exposed during their growth phase to different concentrations of the toxicant for a few days (Naumann et al. 2007; Mkandawire et al. 2014; Ziegler et al. 2016; Farkas and Booth 2017). However, while the ISO guidelines are designed to determine how plant models respond to toxicants and mixtures contained in water samples, treated municipal wastewater, and industrial effluents (i.e., environmental samples), OECD tests focus on assessing the toxicological effects of specific substances and chemical products on plants (Fomin et al. 2000).

The plant-based ecotoxicological tests available refer to standardized guidelines which differ according to the type of freshwater plant organisms used. In particular, guidelines were formulated for microalgae (OECD 201 2011; ISO 8692 2012), rootless macrophytes (ISO 20079 2005; OECD 221 2006), and rooted macrophytes (ISO 16191 2013; OECD 238-239 2014a, b).

With regard to microalgae, the OECD formulated specific guidelines in 1984 (OECD 201 1984) which were then updated in 2011, both by revising the data analysis procedures and by expanding the set of microalgal species used as biological models which the toxicity of various contaminants should be tested on. According to these guidelines, the effects of the contaminant on the growth of certain microalgae, such as the unicellular green algae *Pseudokirchneriella subcapitata* and *Desmodesmus subspicatus* (= *Scenedesmus subspicatus*) and the diatom *Navicula pelliculosa*, are determined within 72 h. The response is evaluated as a function of the exposure concentration in comparison with the average growth of replicate, unexposed control cultures. The ISO guidelines designed for this plant group, initially adopted in 1989 and then revised in 2012 (ISO 8692 1989, 2012), show procedures that are very similar to the OECD ones, except for the type of growth medium and the used microalgal species that only refer to *Pseudokirchneriella subcapitata* and *Desmodesmus subspicatus*.

The OECD guidelines 221 (2006) and ISO 20079 (2005) use floating rootless flowering plants of the genus *Lemna* (*L. minor*, *L. gibba* and only *L. minor*, respectively) as biological models. In both cases, the toxicological effects of the contaminant on the plant growth are quantified over 7 days by measuring the number of fronds, the total surface of the fronds, and the dry or fresh weight.

The Society of Environmental Toxicology and Chemistry (SETAC) highlighted that some toxicants had no effect on algae and rootless plants, probably due to their mode of action or because they typically occur as precipitates in sediments, thus leading to plant exposure to the toxicants only through root uptake (Maltby et al. 2009). This observation, which was also confirmed by a series of experimental studies (Knauer et al. 2006; Arts et al. 2008; Maltby et al. 2010), highlighted the need to develop guidelines that could assess the risk of a contaminant even on rooted plants. The formulation of the ISO guidelines 16191 (2013) first, and then to the OECD 238 and 239 (2014a, b), which provide for use and absence of sediment during the tests respectively, responds to this need using rooted plants of the genus *Myriophyllum* (*M. aquaticum* in the ISO and *M. spicatum* in the OECD); these plants have proved to be among the most suitable aquatic plants when it comes to performing tests that also analyze sediment contamination (Knauer et al. 2008; Kubitza and Dohmen 2008; Maltby et al. 2009). The phytotoxicological effects (variations in shoot length and in fresh or dry weight, stem alterations in terms of chlorosis, necrosis, and malformations) of a chemical contaminant on exposed *Myriophyllum* specimens are evaluated over a longer period of time (i.e., 14 days) than those adopted in the other guidelines. It should be emphasized that the need to carry out tests on rooted plants does not arise with the aim of replacing other phytotoxicological tests to monitor the ecotoxicological status of freshwaters, but rather for integrating them and for having a more exhaustive assessment of the risks which the different aquatic plants can be exposed to, and that also concern the entire environment in which they live.

### Critical issues in standardized plant-based ecotoxicological tests

Some critical issues emerged from the literature about the protocols proposed by the standardized ISO and OECD guidelines. Methodologies and conditions are not always suitable for the optimal application of ecotoxicological tests based on freshwater plants (e.g., Cairns and Niederlehner, 1995; Navarro et al. 2002; Gubbins et al. 2011; Pereira et al. 2018; Ding et al. 2019) mainly due to the following limitations:

i. *Ecosystem complexity*. Most of these guidelines refer to experimental tests carried out exclusively under controlled laboratory conditions; this obviously involves some problems (e.g., short time frame, small scale, lack of synergic effects, and complexity of interaction found in nature) that restrict the capability to extrapolate the real status of a natural system from controlled experiments (Carpenter 1996; Petersen et al. 1997; Schindler 1998). Therefore, the outputs obtained do not fully reflect the actual harmful effects of tested contaminants on plants in nature. For example, the toxic effects of silver nanoparticles (AgNPs) on *Lemna minor* (Ding et al. 2019) are mitigated by the presence of natural suspended substances such as humic acids (HA). HA appear to reduce the absorption capacity of AgNPs in *Lemna*, therefore limiting the phytotoxical effects in the plant.
ii. *Exposition time span*. Several studies (e.g., Gubbins et al. 2011; Pereira et al. 2018) showed how the extension of exposure compared to the time required by the standardized guidelines (for example from 7 to 14 days) can amplify the intensity of the response when testing plant models, providing additional evidence on the contaminant toxicity.
iii. *Growth medium tipology*. The use of an undiluted standard growth medium, as proposed by the standardized guidelines, can interfere with the tested toxicants. For example, a study on the toxic effect of AgNPs on *Lemna minor* by Gubbins et al. (2011) found that AgNPs had a different impact on the aquatic plant depending on whether a diluted or concentrated growth medium was used in the experiment. This was due to the interaction of the toxicant with the medium, and specifically to how the aggregation and sedimentation properties of the toxicant were modified. Once this toxicant-medium interaction was observed, Gubbins et al. (2011) used a 100-fold dilution of the growth medium to ensure the growth of the biomodel used, while reducing AgNP-medium aggregation. This evidence suggests that similar problems could also occur in other standardized phytotoxicological tests and, therefore, it becomes mandatory to perform preliminary tests to verify any interactions between toxicant and medium.
iv. *Endpoints*. The standardized tests only make use of endpoints which in some cases are proved to be reductive. In particular, the endpoints concern the vegetative growth after plant being exposed to contaminants, quantitatively assessing the produced biomass (total leaf surface, shoot length, fresh and/or dry weight) or qualitatively observing some responses, such as chlorosis, necrosis, and/or morphological deformations during the growth. Some studies (Navarro et al. 2002; Alkimin et al. 2019; Dumont et al. 2019) showed that it is necessary to evaluate the effects on plants by extending the assessment to more sensitive plant endpoints. The most frequently used non-standard endpoints include the content of chlorophyll *a*, *b*, total, carotenoids, anthocyanins, and malondialdehyde, the latter being considered a useful biomarker of oxidative damage in plant tissues (Bailly et al. 1996).

### Toxicants tested in freshwater plant-based ecotoxicological tests

**Fig. 2:**
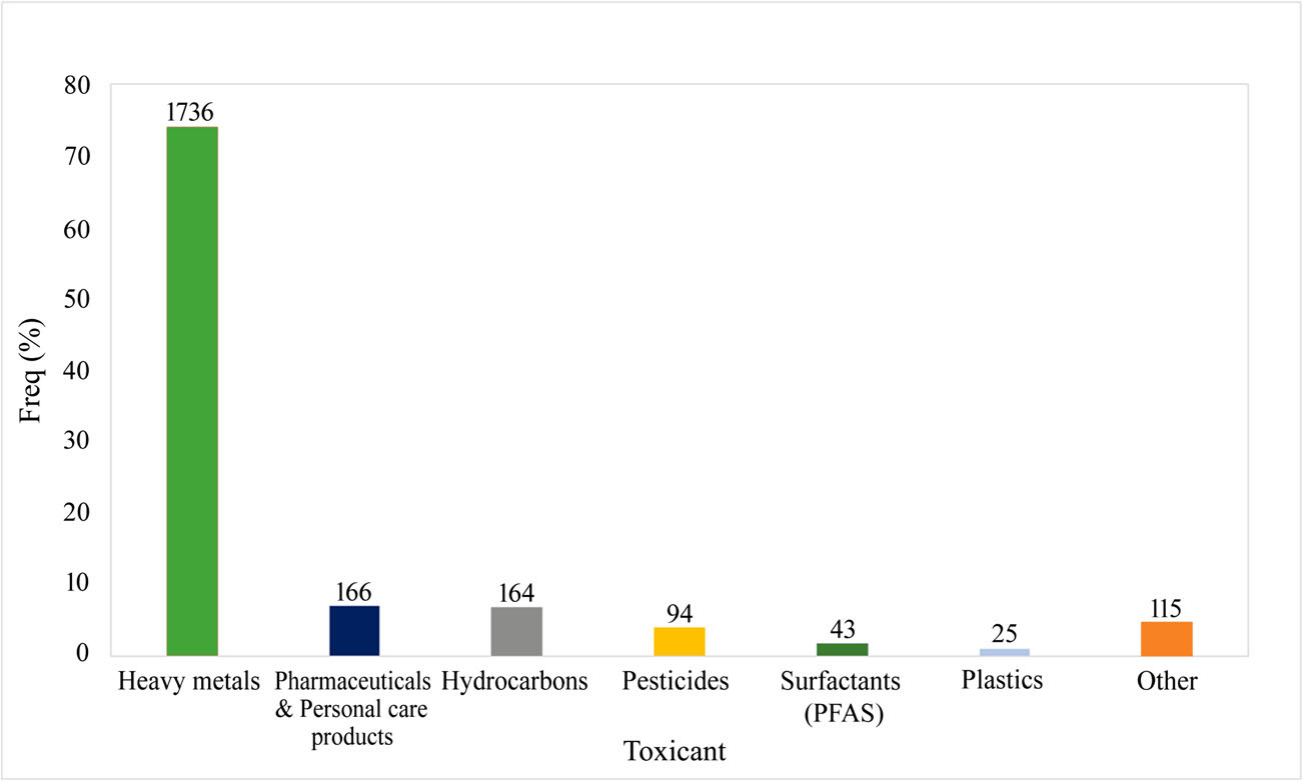
Frequency (%) of toxicants investigated in plant-based ecotoxicological tests. Data extracted and elaborated by ECOTOX (EPA 2000), with the exception of plastics being available in other digital information sources (Scopus, Web of Science, Google Scholar). “Other” includes more sporadic contaminants (< 1%), such as explosive products, major ions (e.g., Ca, Mg, K, Na), and perchlorates

Based on the ecotoxicological studies available in ECOTOX database (EPA 2020), it emerged that different categories of toxicants were tested (Fig. 2) for evaluating toxic effects on plant organisms and finding the limit concentrations (IC_50_ - half maximal inhibitory concentration) to avoid health risks. Among the various toxicants, heavy metals are the most analyzed (74%), followed by pharmaceutical and personal care products and hydrocarbons (around 7%). Recently, other emerging contaminants, such as plastics, attracted the attention of researchers in assessing their toxicity also in freshwaters (van Sebille et al. 2015; Ma et al. 2019), but only few studies investigated toxicity responses in freshwater plants (Kalcíková et al. 2017; Yi et al. 2019).

The methods for testing the various toxicants in plant-based ecotoxicological tests differ slightly in design but basically utilize easily cultured plant organisms which are exposed to different toxicant concentrations for a time range that can vary from few hours to several days. Generally, the test organism grows in a nutrient-enriched medium which is often diluted. At the end of the exposition, the toxicant effects on the biological model are evaluated by analyzing various endpoints that can be both at sub-individual (photosynthesis inhibition, variation in enzymatic activities, chlorophyll fluorescence, pigment content) and individual level (growth rate, chlorosis, leaf number, frond area, fresh weight, leaf and root anatomy).

In Table 2 are summarized the experimental conditions (toxicant concentrations, exposition time (h), growth media, plant endpoints, test plant species) that are generally adopted in freshwater phytotoxicity tests. This data was extracted by some of the more representative studies regarding the different toxicants analyzed. Below, the main toxicants tested in freshwater plant-based ecotoxicological tests are listed.

**Table 2:** The experimental conditions (toxicant concentration, plant detrimental toxicant concentration, exposition time, growth media, plant endpoint analyzed, test plant species) that are generally adopted in freshwater phytotoxicity tests are reported here. The data are extracted by some of the more representative studies regarding the various analyzed contaminants

Reference	Toxicant concentration	Plant detrimental toxicant concentration	Exposition time (h)	Growth media	Plant endpoint	Test species
Heavy metal						
Dumont et al. (2019)	0.1, 0.5, 1, 2, 3 mg/L	EC50 = 0.237, 0.128 mg/L	288	Modified OECD media	Growth rate (phytomass production, length/frond)	*Myriophyllum spicatum*
Gubbuns et al. (2011)	5, 10, 20, 40, 80, 160 μg/L	Time and increasing concentration dependent	168, 336	OECD media	Growth rate, frond number, dry weight	*Lemna minor*
Razinger et al. (2007)	0.25, 0.5, 1, 2, 5, 10 μM	Time and increasing concentration dependent	24	Modified Steinberg	Enzymatic activity, chlorophyll fluorescence	*Lemna gibba*
Pharmaceuticals						
Alkimin et al. (2019)	20, 100, 125 μg/L	> 20 μg/L	96	Modified Steinberg	Enzymatic activity, pigment analyses, chlorophyll fluorescence	*Lemna* spp.
Godoy et al. (2018)	6.2, 12.5, 25, 50, 100, 200, 400 mg/L	EC50 = 57.1 mg/L	168	Steinberg	Frond number, frond area, fresh weight	*Lemna minor*
Brain et al. (2004a, b)	0.044, 0.608, 2.664, 24.538 mol/L	Increasing concentration dependent	168	Modified Andrews	Root length, wet weight, dry weight, root number, longest root, node number, plant length, pigment analyses	*Myriophyllum sibiricum*
Hydrocarbons						
Pokora et al. (2010)	0.25 mg/L	0.25 mg/L	24	Bristol	Photosynthetic activity, enzymatic activity	*Desmodesmus* spp.
Mallakin et al. (2002)	0-10 μg/mL	Increasing concentration dependent	168	Half-strength Hutner	Inhibition of photosynthetic activity	*Lemna gibba*
Pesticides						
Ribeiro et al. (2019)	111, 333 μg/L	Time and increasing concentration dependent	168, 336, 504, 672	Hoagland	Fresh matter, leaf anatomy	*Eichhornia crassipes*
Fernández-Naveira et al. (2016)	0.1, 0.25, 0.5, 1, 2 μM	Time and increasing concentration dependent	24, 48, 72, 96	TAP (tris-acetate phosphate) medium	growth rate, dry weight, photosynthetic pigments, protein contents, enzymatic activity	*Chlamydomonas reinhardtii*
Surfactants						
Pietrini et al. (2019)	2, 20, 200 μg/L	No toxic effect, phytoaccumulation	168	Hoagland	Growth rate, frond area, frond number, chlorophyll fluorescence	*Lemna minor*
Boudreau et al. (2003)	12.5, 25, 50, 100, 200, 400 mg/L	IC50 = 81.6, 88.1 mg/L	96	Bristol	Cell density, chlorophyll content	*Chlorella vulgaris*
Plastics						
Kalčíková et al. (2017)	10, 50, 100 mg/L	≥ 10 mg/L	168	Steinberg	Growth rate, chlorophyll content, root length, root cell viability	*Lemna minor*
Besseling et al. (2014)	44-1100 mg NP_s_/L	Increasing concentration dependent	72	WC medium	Growth rate, photosynthetic efficiency	*Scenedesmus obliquus*

#### Heavy metal

Once heavy metals are released into aquatic environment, they can be absorbed by plants (Costa et al. 2018; Xue et al. 2018) and then transferred to animals thereby bioaccumulating along the food chain (Sofyan et al. 2006), turning into a high risk to the ecosystem safety as well as to human health (Sinnett et al. 2010). Heavy metals occur ubiquitously in natural systems in different concentrations and chemical forms, which in turn determine their transport efficiency, bioaccumulation pathway and, thus, toxicity in plants (Wu et al. 2005; Zhao et al. 2015; Xue et al. 2018).

From the literature, it is clear that most phytotoxicological studies focuses their attention on the effects of heavy metals on plants (Supplementary Table 1). Furthermore, it emerges that the most frequently tested heavy metals are copper (Cu), cadmium (Cd), chromium (Cr), and zinc (Zn), while the plant species that are most widely used to test their effects are listed in Supplementary Table 1. The most commonly detected responses by these species in the presence of heavy metals are: reduction in the growth rate, increase in the rate of necrosis and chlorosis, oxidative stress and photosynthesis inhibition (Monferran et al. 2009; Razinger et al. 2010; Upadhyay et al. 2011; Corcoll et al. 2012), increase in the content of malondialdehyde (e.g., Das et al. 2016; Decou et al. 2018), and antioxidant responses (Gonçalves et al. 2019; Li et al. 2018).

#### Pharmaceutical and personal care products

Of particular environmental relevance are the pharmaceutical and personal care products, increasingly used in human and veterinary medicine, that show properties of environmental persistence and biological activity, especially considering long-term exposure (Fent et al. 2006). These are properties, among others, that make these products a potential risk for biocenoses and environmental health once they are released into the aquatic environment. In addition, several studies demonstrated that drug residues in treated wastewater and surface water are widespread (e.g., Brain et al. 2004a, b; Fent et al. 2006; Küster and Adler 2014; Godoy et al. 2018; Grenni et al. 2019) and once released in the environment they may have adverse effects on exposed biocenoses (Küster and Adler 2014; Alkimin et al. 2019).

Although studies on the effects of pharmaceutical products on plants were carried out, they mostly focused on few plant models, in particular on unicellular algae of the genera *Chlorella* and *Pseusokirchneriella*, and free-floating flowering plants of the genus *Lemna* (Supplementary Table 2). The most common effects of these contaminants on plants are activation of CAT hydrolytic activity (Alkimin et al. 2019), decrease of phytomass (Brain et al. 2004a, b), and plant growth inhibition (Halling-Sorensen et al. 1998; Godoy et al. 2018).

#### Hydrocarbons

Several types of substances fall into this category of toxicants, including mainly polycyclic aromatic hydrocarbons (PAHs), and nitrogen, sulfur, or oxygen heterocyclic aromatic hydrocarbons (NSO-HETs).

Over the last 40 years, the interest about hydrocarbons focused mainly upon PAHs (Achten and Andersson 2015) based on a priority list of 16 PAHs established by EPA (Andersson and Achten 2015; Keith 2015). However, few studies on the toxic effects of PAHs on freshwater plants were actually carried out, probably because most PAHs are not acutely toxic under laboratory conditions. In natural conditions, however, in presence of relevant solar radiation, a number of PAHs were found to be acutely toxic to aquatic organisms at concentrations that were similar to those tested in laboratory (Landrum et al. 1984; Oris et al. 1984), thus highlighting the importance of carrying out toxicological analyses in experimental conditions that are as similar as possible to those found in field. Also, the knowledge on ecotoxicity of NSO-HETs to aquatic plants is still rather scarce (Brendel et al. 2018).

As for negative effects of hydrocarbons on aquatic plants, the available studies (Supplementary Table 3) show that hydrocarbons or their degradation products exhibit toxicity to some microalgae at low concentrations in the mg/L range as genotoxic and mutagenic agents (Eisentraeger et al. 2008). The most common responses concern plant growth inhibition, and alteration in photosynthesis and respiration (Marwood et al. 1999; Aronsson and Ekelund 2005; Grote et al. 2005; Engel et al. 2015; Bi et al. 2016; Kottuparambil and Park 2019), such as it was also highlighted by the study of El-Dib et al. (2001), where the increase in hydrocarbon concentration corresponded to a decrease in plant growth rate and chlorophyll content.

#### Pesticides

The increasing use of pesticides (herbicides, fungicides, insecticides), linked to the increase in intensive agricultural practices, contributed to the progressive contamination of the environment and surface waters (Dumont et al. 2019). These chemicals enter into aquatic ecosystems through spraying and drifting, soil leaching, surface runoff, and accidental spills (Ma et al. 2008), and once entered, their adverse effects on non-target plants are of particular concern because of their ever-increasing periodical release (van Der Brink and Ter Baak 1999). When pesticides reach the aquatic ecosystem, their toxic potential can vary depending on their solubility and persistence in water, as well as their potential to be absorbed by aquatic plants (Neto et al. 2017; Salazar-Ledesma et al. 2018; Ribeiro et al. 2019).

In some studies (Supplementary Table 4) it was observed that among the most common effects of pesticides on aquatic plants there were a lower production of phytomass and alteration of the leaf structure in vascular plants (Ribeiro et al. 2019), and a partial or complete growth inhibition and alteration of the metabolic pathways after 96 h of exposure in some algal species (Fernández-Naveira et al. 2016; Flood et al. 2018).

#### Surfactants

Surfactants are mobile organic compounds released into the environment in great volume, and for this reason, strategies to enhance their degradation are of great interest. Some surfactants are metabolically and chemically inert, resisting to both abiotic degradation (Sharpe 1971; Boudreau et al. 2003) and biotic (Remde and Debus 1996; Key et al. 1998), thus becoming persistent and bioaccumulable in the environment, such as the non-biodegradable fluorosurfactant, perfluorooctanoic acid (PFOA). Perfluoroalkyls (PFAS) are among the most used surfactants in a wide range of industrial and consumer products (Prevedouros et al. 2006; Buck et al. 2011) and, specifically, the most used and studied molecules are perfluorooctanoic acid (PFOA) and perfluorooctanesulfonic acid (PFOS).

Uptake, metabolism and toxicity of PFAS in terrestrial plants were studied (Wen et al. 2013; Blaine et al. 2014; Garcia-Valcarcel et al. 2014; Krippner et al. 2014), while their effects on aquatic plants are still poorly known (Supplementary Table 5). In this regard, Boudreau et al. (2003) and McCarthy et al. (2017) tested the effects of PFAS on the growth of two different *Lemna* species, *L. minor* and *L. gibba*, highlighting that the latter showed a higher sensitivity to PFOS as it was more strongly inhibited in growth; however, it should be noted that the concentration that produced the toxic effect was higher (mg/L) than the one recorded in the surveys on the natural environment (ng/L). Conversely, Pietrini et al. (2019) demonstrated that at PFAS concentrations close to those actually detected in nature, inhibitory effects in biometric and physiological descriptors were not found in *L. minor*. However, the study highlights the important role of the plant species as primary producers and, therefore, their potential capability to bioaccumulate these substances in their tissues, potentially triggering biomagnification phenomena along the trophic chains.

Regardless of the tested concentrations of surfactants, the most frequently encountered effects in aquatic plants as response to exposition to this toxicant category are growth inhibition, chlorosis, necrosis, and reduction in the number of leaves, while one aspect that does not seem to be affected is the chlorophyll content (Boudreau et al. 2003).

#### Plastics

As contaminants, plastics are a scientific and social emerging concern for the conservation of the environment in which they are released (Ma et al. 2019). Although the problem of plastic pollution has initially exploded in marine ecosystems (e.g., Barnes et al. 2009; Browne et al. 2011; Cole et al. 2011; Arossa et al. 2019), some recent studies underlined how it is a source of equally serious risk to freshwater ecosystems (Zbyszewski and Corcoran 2011; Zbyszewski et al. 2014; Koelmans et al. 2015; Mattsson et al. 2015; van Sebille et al. 2015).

To date, ecotoxicological studies testing the effects of micro- or nano-plastics on freshwater plants are still very scarce. Very few plant species were tested with these contaminants, including microalgal species of the genus *Chlorella* and *Scenedesmus* and flowering plants such as *Lemna minor* and *Myriophyllum spicatum* (Supplementary Table 6). However, the limited available literature shows that the phytotoxicological effects of the most commonly encountered plastics include photosynthesis inhibition and sprout and root growth (Kalcíková et al. 2017; Bosker et al. 2019; Dovidat et al. 2019; van Weert et al. 2019), as micro- and nanoplastic particles adsorbed on external plant tissues form physical blocks to light and air by hindering photosynthesis and respiration activities (Bhattacharya et al. 2010; Besseling et al. 2014; Kalčíková et al. 2017; Mateos-Cárdenas et al. 2019; Ma et al. 2019; van Weert et al. 2019; Yi et al. 2019). However, many of these studies showed that generally plant species are only affected when the concentrations of micro- and nanoplastics are higher than those recorded in nature (Mateos-Cárdenas et al. 2019; van Weert et al. 2019).

## Conclusion

The ubiquitous distribution of toxicants in freshwater ecosystems makes a wide range of aquatic communities threatened by their exposure, inducing a variety of negative effects at diverse trophic levels, starting from plant organisms as primary producers, to consumers, to superpredators (including humans). To date, however, it appears that only few investigations are addressing the processes of toxicant transfer along the trophic chains so far, and consequently all the implications for human health arising from consumption of contaminated aquatic organisms (Wang et al. 2019).

The analysis of studies on the effects of toxicants on freshwater plants highlighted that most of them were carried out in controlled laboratory conditions, which significantly reduces the ecological relevance, that is, the possibility of adequately projecting plant responses in nature. Although many of the toxicological investigations on freshwaters pass off as ecotoxicological studies, most of them actually deviate from the main purpose of ecotoxicological research; such research, indeed, consists in analyzing the accumulation, transport, transformation, and degradation of contaminants once they are introduced into the environment, and their effect on the various aquatic biocenoses, and on humans as a consequence of direct/indirect use of contaminated resources (Cairns and Niederlehner 1993; Forbes and Forbes 1994). The fact is that most of these “ecotoxicological” studies recall classical toxicology investigations that analyze the effects of contaminants on organisms through laboratory tests, without therefore assessing them in a real context of environmental complexity that includes a variety of pollutants and their interactions with organisms (e.g., Wright and Welbourn 2002; Caussy et al. 2003; Peijnenburg and Jager 2003; Dirilgen 2011; Mestankova et al. 2011; Radić et al. 2011). This criticism also emerges by analyzing the ECOTOX database (EPA 2020), filtering the available data under the item “test site”; indeed, it emerges that over 90% of the plant-based ecotoxicological studies were carried out under strictly laboratory conditions (93%), 5% in controlled field, and only 2% in natural field.

Another criticism is that the ecotoxicological studies prove to be focused mainly on aquatic animal organisms, fact that reveals the poor monitoring of ecotoxicological effects of toxicants on aquatic plants despite their ecological importance and fundamental functions that play at ecosystem level. In addition, among aquatic plants, particular attention should be given to those of freshwater environments whose integrity and conservation are at risk, and they even more threatened than those in marine environments (Pang et al. 2017; Cañedo-Argüelles et al. 2019; Ma et al. 2019). It should be noted that many of the contaminants found in marine habitats are transported by rivers (Besseling et al. 2014; Rech et al. 2014; dos Santos et al. 2018), which thus become the main sources of pollution in seas and oceans.

 In light of the critical issues emerging from this investigation, the following suggestions are provided for future phytotoxicological studies in freshwater ecosystems:

- to increase the ecological relevance of ecotoxicological studies by reproducing as frequently as possible the real environmental conditions; in fact, in order to establish the real toxicity effects of a pollutant in a waterbody, after having carried out laboratory tests according to standardized protocols, it is necessary to design tests that faithfully portray the mechanisms and the ecosystem complexity, in order to obtain more reliable responses with respect to what actually occurs in the environment;
- to use toxicant concentrations that are environmentally relevant; the use of higher toxicant concentrations than those recorded in nature is very useful to define the toxicity levels, but does not help in (i) understanding the real effects of the toxicant within the ecosystem, and (ii) proposing the right mitigating measures;
- to expand both the range of contaminants to be tested and the spectrum of aquatic plant species to be used as biological models for biomonitoring the toxicological effects of contaminants released in the environment. In fact, in order to have a more exhaustive understanding of the toxicological effects of a substance on the various biocenoses and the entire ecosystem, it is necessary to direct the investigations towards an integrated biological approach, carrying out parallel tests on both animals and plants; this approach follows the principle that analyzing different biological groups can provide more information than analyzing one group only, by showing different sensitivities and, therefore, different biological responses to a certain contaminant;
- to extend the exposure time in order to obtain more amplified and, thus, more identifiable plant responses. Although the extension of exposure time would lead to a longer experimentation compared to other no-plant organisms, such as aquatic invertebrates and fish (from 48 to 96 h), the identification of toxic effects on plants (i.e., on primary producers) would imply that the risk of contamination or accumulation along the trophic chain could be identified at an early stage; therefore, plants can be used as early warning systems (EWSs), whose monitoring becomes essential to promptly intervene in case of environmental contamination;
- to hypothesize, in the case of tests involving the use of a growth medium, preliminary tests to verify possible interactions and/or aggregations between tested toxicants and the growth medium;
- to expand the range of plant endpoints; for example, to consider those biomarker responses that may be more specific than those endpoints analysed in the standardized guidelines. The identification of specific responses would increase the possibility of identifying more precisely the presence of a certain contaminant;
- to differentiate ecotoxicological studies according to the type of waterbody (e.g., river, lake, pond), taking into account the dominant plant communities within it.

## Supplementary Information

ESM 1 (XLSX 18.2 kb)

ESM 2 (XLSX 11.2 kb)

ESM 3 (XLSX 12.2 kb)

ESM 4 (XLSX 12.2 kb)

ESM 5 (XLSX 11.1 kb)

ESM 6 (XLSX 11.3 kb)

## Data Availability

All data generated or analyzed during this study are included in this published article.
